# Supplementary material for: Coping with the mental health impact of COVID–19: A study protocol for a multinational longitudinal study on coping and resilience during the COVID-19 pandemic
Source: PLoS One. 2023 May 18;18(5):e0285803. doi: 10.1371/journal.pone.0285803 (PMC10194934; doi:10.1371/journal.pone.0285803)
Supplement: S1 Table — (DOCX) [file pone.0285803.s001.docx]

**S1 Table.** Instruments used in COPERS

| **Variable** | **Instrument** | **Author(s), year** | **Doi** |
| --- | --- | --- | --- |
| Alcohol consumptions | Alcohol Use Disorders Identification Test Concise  (AUDIT-C) | Bush, Kivlahan, McDonell, Fihn, Bradley, 1998 | 10.1001/archinte.158.16.1789 |
| Anxiety | Generalized Anxiety Disorder-7 | Spitzer, Kroenke, Williams, Löwe, 2006 | 10.1001/archinte.166.10.1092 |
| Depressive Symptoms | Patient Health Questionnaire-9 (PHQ-9) | Kroenke, Spitzer, Williams, 2001 | 10.1046/j.1525-1497.2001.016009606.x |
| Loneliness | UCLA loneliness scale | Russell, Peplau, Cutrona, 1980 | 10.1037//0022-3514.39.3.472 |
| Resilience | Connor-Davidson Resilience Scale (CD-RISC) | Connor, Davidson, 2003 | 10.1002/da.10113 |
| Resilience | Brief Resilience Scale (BRS) | Smith, Dale, Wiggins, Tooley, Christopher, Bernard, 2008 | 10.1080/10705500802222972 |
| Social capital | World Bank: Measuring Social Capital: An Integrated Questionnaire | Grootaert, Narayan, Nyhan Jones, Woolcock, 2004 | http://hdl.handle.net/10986/15033 |
| Stress | Impact of Event Scale-Revised | Weiss, 2007 | 10.1007/978-0-387-70990-1_10 |
| Suicidal ideation | Item-9 of the PHQ-9 | Kroenke, Spitzer, Williams, 2002 | 10.1001/archinte.166.10.1092 |
