# Supplementary material for: Coping with the mental health impact of COVID–19: A study protocol for a multinational longitudinal study on coping and resilience during the COVID-19 pandemic
Source: PLoS One. 2023 May 18;18(5):e0285803. doi: 10.1371/journal.pone.0285803 (PMC10194934; doi:10.1371/journal.pone.0285803)
Supplement: S1 File — (PDF) [file pone.0285803.s002.pdf]

University of Applied Sciences
HOCHSCHULE EMDEN·LEER

**Der Präsident**
Vizepräsident für Forschung & Wissenstransfer

HS Emden/Leer ▪ Constantiaplatz 4 ▪ 26723 Emden

**Auskunft erteilt**

Name: Prof. Dr. Sven Steinigeweg
E-Mail: vp.forschung-transfer@hs-emden-leer.de
Tel.: 04921/807-1007
Fax: 04921/807-1003

Frau Prof.
Dr. Jutta Lindert
FB Soziale Arbeit und Gesundheit

Ihr Zeichen
Ihre Nachricht (Datum)

(Bei Antwort angeben)
Mein Zeichen
st

Emden, 1. Oktober 2020

**Genehmigung Ihres Antrags „COPERS“**

Sehr geehrte Frau Prof. Lindert,

gerne teile ich Ihnen mit, dass die Kommission für Folgenabschätzung und Ethik Ihren Antrag genehmigt hat.

Ich wünsche Ihrem Vorhaben viel Erfolg und verbleibe
mit freundlichen Grüßen

Prof. Dr. Sven Steinigeweg
Vizepräsident für Forschung und Wissenstransfer
