# Supplementary material for: Coping with the mental health impact of COVID–19: A study protocol for a multinational longitudinal study on coping and resilience during the COVID-19 pandemic
Source: PLoS One. 2023 May 18;18(5):e0285803. doi: 10.1371/journal.pone.0285803 (PMC10194934; doi:10.1371/journal.pone.0285803)
Supplement: S3 File — (PDF) [file pone.0285803.s004.pdf]

POLITECNICO
MILANO 1863

Milan, on 12/11/2020

Opinion n. 31/2020

RESEARCH SERVICE
RESEARCH AND
INNOVATION

OBJECT: Research Ethical Committee Opinion. Project: *"Coping with COVID" (COPERS)"*. Scientific coordinator: Prof. Stefano Capolongo.

The Research Ethical Committee,

- given the request for opinion of 02/11/2020 concerning the aforementioned project;
- given the audit of the meeting of 05/11/2020;
- examined all documents;

issues the following opinion:

POSITIVE

The President
(Prof. Carlo Ghezzi)

Politecnico di Milano
Research Service
Piazza Leonardo da Vinci, 32
20133 Milan
Phone: 02 2399 4345
Fax 02 2399 2575
www.polimi.it
