# Supplementary material for: Coping with the mental health impact of COVID–19: A study protocol for a multinational longitudinal study on coping and resilience during the COVID-19 pandemic
Source: PLoS One. 2023 May 18;18(5):e0285803. doi: 10.1371/journal.pone.0285803 (PMC10194934; doi:10.1371/journal.pone.0285803)
Supplement: S4 File — (PDF) [file pone.0285803.s005.pdf]

MINISTRY OF EDUCATION AND RESEARCH

UNIVERSITY OF MEDICINE AND PHARMACY OF CRAIOVA
Committee of Ethics and Academic and Scientific Deontology

No. 147 / 07.12.2020

Ethical Approval of Research Project

For the study (research project)

COPING WITH COVID (COPERS)

***Study coordinator**:* Prof. Jutta Lindert, University of Applied Sciences Emden/Leer, Germany

***Research team**:* Germany, Prof. Jutta Lindert, University of Applied Sciences Emden/Leer • Albania, Dr. Edvaldo Bogaterej, Sapienza University of Rome, Italy • Belgium, Prof. Vincent Lorant University of Louvain • Bulgaria, Prof. Vladimir Nakov, National Center of Public Health and Analysis Bulgaria • Croatia, Prof. Danijela Štimac Grbić • Italy, Prof. Mauro Carta, University of Cagliari • Israel, Prof. Haim Knobler, Hadassah University • Lithuania, Prof. Marija Jakubaskiene, Vilnius University • Malta, Prof. John Cachia, Office of the Commissioner For Mental Health • Norway, Arnstein Mykletun Norwegian Institute of Public Health, Oslo • Romania, Lecturer Mihail Cristian Pirlog, University of Medicine and Pharmacy of Craiova • Serbia, Prof. Marija Jevtic, University of Novi Sad • Slovenia, Prof. Matej Vinko, National Institute of Public Health of Slovenia • Spain, Prof. Jordi Alonso, IMIM Hospital del Mar Medical Research Institute • UK, Shari McDavid.

We confirm that the guidelines of the Ethical University Code of The University of Medicine and Pharmacy from Craiova were consulted and all ethical issues and implications related to the above project were considered. The study procedures were followed in accordance with these guidelines.

Ethical principles were followed underlying the Declaration of Helsinki and the University Code of Ethics on the proper conduct of research, together with the codes of practice established by the medical ethics code.

Ethical Approval:	Yes ☑	No ☐	
Date: 07.12.2020			

Signature of Ethics Committee Chairman,
Ionică-Daniel Vîlcea, PhD., MD.

2 Petru Rares Street, 200349, Craiova, Dolj, ROMANIA
Phone: + 40 351 443522; Fax: +40 251 593077; E-mail: etica@webmail.umfcv.ro
