# Supplementary material for: Coping with the mental health impact of COVID–19: A study protocol for a multinational longitudinal study on coping and resilience during the COVID-19 pandemic
Source: PLoS One. 2023 May 18;18(5):e0285803. doi: 10.1371/journal.pone.0285803 (PMC10194934; doi:10.1371/journal.pone.0285803)
Supplement: S2 File — (PDF) [file pone.0285803.s003.pdf]

Cliniques universitaires de Bruxelles
Route de Lennik 808
B - 1070 Bruxelles
T +32 (0)2 555 31 11
M contact@erasme.ulb.ac.be
S www.erasme.ulb.ac.be

Hôpital Erasme

ULB

Dr. Simone Pietro MARCHINI
Service de Psychiatrie

Hôpital Erasme

Brussels, April 6, 2021

Comité d'Ethique hospitalo-facultaire Erasme - ULB
(021 / 406)

Secrétariat
T 02 555 37 07
02 555 37 11
F 02 555 46 20
M comite.ethique@erasme.ulb.ac.be
www.erasme.ulb.ac.be/ethique

Dear Dr. MARCHINI,

Please find further on the answer of the Ethics Committee concerning the below mentioned study.

ETHICS COMMITTEE APPROVAL

We, undersigned, "Board Members" of Ethics Committee Erasme Hospital
808, route de Lennik
B-1070 Brussels, Belgium

confirm that the documents submitted for approval were initially examined on 23/02/2021 (dd/mm/yyyy).

After examination of the answers to the comments and of the new version of the information sheet and consent form received the 06/04/2021, the Ethics Committee gave its agreement on 07/04/2021 to the following documents:

- Protocol **(References : P2021/142 / B4062021000074)** entitled: ***"L'étude COPERS : faire face à la situation COVID-19."*** (Your Ref.: Version 1.1, dated March 5, 2021)
- French Patient Information Sheet: Version 1.2, April 6, 2021 and French Informed Consent Form: Version 1.2, April 6, 2021

The following document(s), included in the submission file, has/have also been reviewed:

- Ethics Committees Application form signed the 03/02/2021
- Certificate of Insurance: Ethias Insurance Erasme - Policy Nr 45.415.046 - Classe Ia, Ib, II, III
- Clinical Trial Agreement: Data transfer agreement : Draft or not yet signed by all the concerned parties
- Advertissement, version 1.1, dated March 5, 2021 (in French)
- CRF
- Patient's Message, Version 1.2, April 6, 2021(in French)
- Questionnaire, Version 1.2, April 6, 2021 (in French)
- Synopsis, version 1.0, dated February 3, 2021 (in English)
- Information's Letter, Version 1.0, dated October 30, 2020 (in English)
- Information's Letter, Version 1.0, dated January 25, 2021 (in French)

We also confirm the suitability of the local investigator and his team and the quality of local facilities.

This agreement is valid from 07/04/2021 till 30/03/2022.

The Committee reminds the investigator of his personal responsibility for this project. We consider **your responsibility**, in accordance with the recommendations of ICH-GCP (E6):

Point 3.3.6.: no subject should be admitted to a trial before the IRB/IEC issues its written approval/favourable opinion of the trial.

Point 3.3.7.: no deviations from, or changes of, the protocol should be initiated without prior written IRB/IEC approval/favourable opinion of an appropriate amendment, except when necessary to eliminate immediate hazards to the subjects or when the change(s) involves only logistical or administrative aspects of the trial (e.g., change of monitor(s), telephone number(s)).

./..

Point 3.3.8.: the investigator should promptly report to the IRB/IEC:

a) Deviations from, or changes of, the protocol to eliminate immediate hazards to the trial subjects.
b) Changes increasing the risk to subjects and/or affecting significantly the conduct of the trial.
c) All adverse drug reactions (ADRs) that are both serious and unexpected (each suspected and unexpected severe adverse reaction (SUSAR) will be transmitted to this Ethics Committee with your appreciation of the relationship between drug and adverse reaction, and the impact on the security of all participants to the assay).
d) New information that may affect adversely the safety of the subjects or the conduct of the trial.

Moreover, annual trial status and **end trial results** will be transmitted to the Committee.

If Dutch speaking participants have to be recruited for this study, it is your responsibility to submit to the Ethics Committee a Dutch version of the information sheet and consent form.

The list of the names and qualifications of the members of the Ethics Committee is added in appendix.

We hereby confirm that this Ethics Committee is organized and operates according to ICH GCP and the applicable law and regulations.

Pr J.-M. BOEYNAEMS
Chairman

Cliniques universitaires de Bruxelles
Route de Lennik 808
B - 1070 Bruxelles
T +32 (0)2 555 31 11
M contact@erasme.ulb.ac.be
S www.erasme.ulb.ac.be

Hôpital Erasme ULB

List of Members EC Erasme-ULB
2020 - 2024

Comité d'Ethique
hospitalo-facultaire
Erasme - ULB
(021 / 406)

Secrétariat
T 02 555 37 07
02 555 37 11
F 02 555 46 20
comite.ethique@erasme.ulb.ac.be
www.erasme.ulb.ac.be/ethique

Président

Pr J.-M. BOEYNAEMS

Bureau

Pr S. GOLDMAN
Pr H. LOUIS
Pr J.-L. VACHIERY
Pr S. BAUDRY
Pr I. LANGER

Consultants

Pr B. BYL
Pr I. DEMEESTERE
Pr A. DEMOLS
Pr Fl. ROUFOSSE
Pr F. VERMEULEN
Pr Ph. PEIGNEUX
Mr B. FONTEYN
Mme Th. LOCOGE

Pr J.-M. **BOEYNAEMS**, Biologist, MD, PhD, Male, Scientist, Affiliated
Pr S. **GOLDMAN**, Neurologist, MD, PhD, Male, Scientist, Affiliated
Pr H. **LOUIS**, Gastroenterologist, MD, PhD, Male, Scientist, Affiliated
Pr J.-L. **VACHIERY**, Cardiologist, MD, Male, Scientist, Affiliated
Pr S. **BAUDRY**, Physiologist, Bsc, PhD, Male, Scientist, Affiliated
Pr I. **LANGER**, Pharmacologist, PharmaD, PhD, Female, Scientist, Affiliated
Mrs B. **ABBOUZ**, Lawyer, Female, Non Scientist, Affiliated
Mrs L. **BENAMMAR**, Hospital Pharmacist, Female, Scientist, Affiliated
Dr J.-C. **BIER**, Neurologist, MD, Male, Scientist, Affiliated
Mr. A. **COUNET**, Member of a Patients Association, Male, Non Scientist, Non Affiliated
Pr N. **D'HAENE**, Pathologist, MD, PhD, Female, Scientist, Affiliated
Mrs L. **DIDENS**, Nurse, Female, Non-Scientist, Affiliated
Mme V. **DUTHIE**, Nurse, Female, Non-Scientist, Affiliated
Pr V. **FONTAINE**, Microbiologist, PhD, Female, Scientist, Affiliated
Mr R. **GIOFFREDI**, Member of a Patients Association, Male, Non Scientist, Non Affiliated
Dr J. **GROSWASSER**, Paediatrician, MD, PhD, Male, Scientist, Non Affiliated
Dr A. **JOOSTEN**, Anesthetist, MD, Male, Scientist, Affiliated
Dr V. **LESAGE**, Geriatist, MD, Female, Scientist, Affiliated
Pr G. **LOAS**, Psychiatrist, MD, PhD, Male, Scientist, Affiliated
Mr M. **MAYER**, Moral Counsel, BSc, PhD, Male, Non Scientist, Affiliated
Dr L. **NOBILE**, Intensivist, MD, Female, Scientist, Affiliated
Mme A. **PETIAU**, Psychologist, Female, Scientist, Affiliated
Dr D. **PIQUARD**, General Practitioner, MD, Male, Scientist, Affiliated
Mr. C. **PLOMTEUX**, Representative Healthy Volunteers, Male, Non Scientist, Non Affiliated
Dr J. **ROUBY**, General Practitioner, MD, Male, Scientist, Non Affiliated
Mme C. **SAUVAGE**, Physiotherapist, BSc, PhD, Female, Scientist, Affiliated
Pr S. **SCHIFFMANN**, Neurobiologist, MD, PhD, Male, Scientist, Affiliated
Mrs S. **SCHOLTES**, Hospital Pharmacist, Female, Scientist, Affiliated
Dr A-L. **TREPANT**, Pathologist, MD, Female, Scientist, Affiliated
Pr Ph. **VAN DE BORNE**, Cardiologist, MD PhD, Male, Scientist, Affiliated (phone call)
Pr C. **VERHOEVEN**, Biostatistician, PhD, Female, Scientist, Affiliated
