# Supplementary material for: Coping with the mental health impact of COVID–19: A study protocol for a multinational longitudinal study on coping and resilience during the COVID-19 pandemic
Source: PLoS One. 2023 May 18;18(5):e0285803. doi: 10.1371/journal.pone.0285803 (PMC10194934; doi:10.1371/journal.pone.0285803)
Supplement: Attachment — Submitted filename: Answer to reviewers_28012022_JL.docx [file pone.0285803.s006.docx]

**Response letter**

Dear Editorial Team and Reviewers,

on behalf of all the co-authors, I thank you very much for your interest in our work and for giving us the chance to revise the manuscript. You have brought up some very good points that will surely improve our manuscript. We have tried to do our best to respond to the points raised and we hope it will now be suitable for publication in your distinguished journal. Please find below your comments (in bold) and our replies.

**Methods section, the name(s) of the ethics committee(s)/institutional review board(s) (IRBs) that approved your protocol, the date of approval, and the approval number. If you did not obtain ethics approval for your protocol, please explain in your manuscript why ethics approval was not necessary and the name(s) of the ethics committee(s)/institutional review board(s) (IRBs) that waived approval of your protocol. More information related to the relevant journal policies is available from: [https://journals.plos.org/plosone/s/criteria-for-publication#loc-6].**

We applied for Ethical approval at the Ethics Committee of the University of Applied Sciences. The Ethics Committee approved the protocol October 1^st^, 2020. Partner universities contacted their respective IRBs, legal regulations requested separate Ethics approval for Romania (Ethical Approval obtained by the Committee of Ethics and Academic and Scientific Deontology, No 147, 7.12.2020), Italy (ethical approval obtained by the Research Ethical Committee, 2/11/2020) and Belgium (Ethics Committee Erasme Hospital, 23/02/2022).

**5. We note you did not include a copy of the original, (signed or stamped) dated IRB/research ethics committee approval letter or waiver for all study sites as an ‘Other’ file. In keeping with our submission guidelines, please upload the approval letter or waiver as an ‘Other’ file. More information related to the relevant journal policies is available from: [https://journals.plos.org/plosone/s/submission-guidelines#loc-guidelines-for-specific-study-types].**

We uploaded the respective files.

**Please provide additional details regarding participant consent. In the ethics statement in the Methods and online submission information, please ensure that you have specified what type you obtained (for instance, written or verbal, and if verbal, how it was documented and witnessed). If your study included minors, state whether you obtained consent from parents or guardians. If the need for consent was waived by the ethics committee, please include this information.**

We specified the details regarding participant consent. Participants gave their written participant consent. In case they did not give their consent, it was impossible to participate in our study.

**Reviewers comments**

**Comment 1. Abstract: The design is not clear to me in the abstract. You talk about a longitudinal study and primary outcomes. Someone wouldn’t know if your study is an intervention study or observational without intervention. Please be clear and indicate what you mean with outcome…maybe you can state what you do in your study at the end of the introduction?! See other protocols as a blueprint.**

Author response: Thank you very much for this comment. To increase clarity, we changed the abstract as follows:

Background: Mental health is challenged due to serious life events such as the COVID-19 pandemic and can differ by the level of resilience. National studies on mental health and resilience of individuals and communities during the pandemic provide heterogeneous results and more data on mental health outcomes and resilience trajectories are needed to better understand the impact of the pandemic on mental health in Europe.

Methods: COPERS (Coping with COVID-19 with Resilience Study) is an observational multinational longitudinal study conducted in eight European countries (Albania, Belgium, Germany, Italy, Lithuania, Romania, Serbia, and Slovenia). Recruitment of participants is based on convenience sampling and data are gathered through an online questionnaire. gathering data on depression, anxiety, stress-related symptoms suicidal ideation and resilience. Resilience is measured with the Brief Resilience Scale and with the Connor-Davidson Resilience Scale. Depression is measured with the Patient Health Questionnaire, Anxiety with the Generalized Anxiety Disorder Scale and stress-related symptoms with the Impact of Event Scale Revised- Suicidal ideation is assessed using item 9 of the PHQ-9. We also consider potential determinants and moderating factors for mental health conditions, including sociodemographic characteristics (e.g., age, gender), social environmental factors (e.g., loneliness, social capital) and coping strategies (e.g., Self-efficacy Belief).

Discussion: To the best of our knowledge, this is the first study to multi-nationally and longitudinally determine mental health outcomes and resilience trajectories in Europe during the COVID-19 pandemic. The results of this study will help to determine mental health conditions during the COVID-19 pandemic across Europe. The findings may benefit pandemic preparedness planning and future evidence-based mental health policies.

**Comment 2. Introduction: Your hypotheses are not precisely derived from your introduction. I do not want to be too picky in this instance for a protocol. Please be more specific in the upcoming publications. There is lots of evidence in the literature already for you hypotheses. We have different findings in different countries for example. Your strength is an assessment in one study. Still, you could cite more specific findings in the introduction.**

Author response: Thank you very much for this comment. While we agree that literature on the association between resilience is emerging, evidence of factors that promote resilience, specifically during the pandemic is lacking. To clarify this, we have adjusted the text and included appropriate references. It reads as follows:

p.4-5: .”…*While research about the importance of resilience for mental health during the pandemic is emerging, it remains unclear, which factors promote or decrease resilience in times of crisis [15–17]. Researchers from the USA and China, for instance, found that individual and family resilience are associated with mental health [16]. However, the authors did not investigate factors that may contribute to resilience. Currently, research examining determinants of resilience is only available from before the COVID-19 pandemic. A pre-pandemic study from Japan, for instance, points towards an association between social capital and resilience in Japan [18]. However, determinants of resilience may be situation-dependent and vary between countries and different population groups. …”*

**Comment 3. Methods. Line 144 ff.: You just mirror the inclusion criteria as exclusion criteria. This is not meant to be exclusion criteria. Please think about actual exclusion criteria that indicate exclusion of participants even though meeting all inclusion criteria. Please read state-of-the-art literature to make sure how exclusion criteria are meant to be. If you don’t have any exclusion criteria – say so!**

Author response: Thank you for highlighting this. We deleted the exclusion criteria and have added the following sentence to clarify that only persons meeting the inclusion criteria can participate: “Persons are not eligible to participate if they do not meet the inclusion criteria.”

**Comment 4. Line 184: Many have been translated does not mean ALL?? What have you done with the rest?**

Author response: Thank you for pointing this out. We have added information on validated translation to each instrument. For instance, for the PHQ-9 the text reads as follows:

*p.8: “The PHQ-9 has been shown to be a valid and reliable measurement tool for depression across European countries [25,27] and has been translated into Dutch, French, German, Italian, Lithuanian, Romanian, and Serbian [28]. To be able to use the PHQ-9 in Albania and Slovenia; the tool was translated using the forward-backward translation approach.”*

**Comment 5: The ordering of primary outcomes and determinants seem arbitrary. Anxiety is a mental health measure just like depression. It is not clear how you come up with the assignment of constructs to the categories of variables. Maybe you can state this somehow in 1-2 sentences somewhere.**

Author response: Thank you very much for this comment. Thank you for your comment regarding anxiety. We have now included anxiety as an outcome in the main text as well as in the abstract.

**Comment 6. Line 260: You do not look for prevalence rates (you would have to diagnose persons clinically for that) but severity of symptoms! Prevalence is a predefined construct in epidemiology. Also, in this sentence anxiety is counted as an outcome all of a sudden.”**

Author response: In epidemiology, biostastics, public health and many other disciplines the prevalence is the proportion of persons in a population who have a particular disease. It is a common measure of the frequency of a particular disease in a population. In the present study we investigate the prevalence of depressive symptoms using a validated and standardized instrument, namely the PHQ-9. The validity and reliability of these measures as been proven in many previous studies. We do not aim to diagnose participants. However, we agree that for clinical psychologists an assessment using a self-reported measures might not be sufficient and an expert opinion. We have therefore adjusted and toned-down the wording throughout the manuscript and now refer to depressive symptoms instead of depression.
