# Supplementary material for: Coping with the mental health impact of COVID–19: A study protocol for a multinational longitudinal study on coping and resilience during the COVID-19 pandemic
Source: PLoS One. 2023 May 18;18(5):e0285803. doi: 10.1371/journal.pone.0285803 (PMC10194934; doi:10.1371/journal.pone.0285803)
Supplement: Attachment — Submitted filename: PLOS comments.docx [file pone.0285803.s007.docx]

The researchers conducted Coping with the mental health impact of COVID–19: a study protocol for a multinational longitudinal study on coping and resilience during the COVID-19 pandemic. I find that the the introduction is clear, I have a few remarks to improve the manuscript:

- Avoid subjective verbs We, I, Our in the document rather use the study, this study…
- Your hypotheses are not clearly written especially the third and fourth hypothesis, try to be more specific.
- Remove this sentence from inclusion criteria “Persons are not eligible to participate if they do not meet the inclusion criteria”
- Be care on some technical terms between multiple and multivariable terms. Re-correct it
- Add more important implications for health (individual and/or public), as well as health care, policy and for other researchers
- Why you impose language limits,
- Try to answer all of the objectives in clear manner since it is important?
- Make your discussion more strong.
- On conclusion part it is expected to an
- Unify the references
